# Genetic Diversity and Dispersal of *Aspergillus fumigatus* in Arctic Soils

**DOI:** 10.3390/genes13010019

**Published:** 2021-12-22

**Authors:** Gregory A. Korfanty, Mykaelah Dixon, Haoran Jia, Heather Yoell, Jianping Xu

**Affiliations:** Department of Biology, McMaster University, 1280 Main Street West, Hamilton, ON L8S 4K1, Canada; korfanga@mcmaster.ca (G.A.K.); dixonm5@mcmaster.ca (M.D.); jiah18@mcmaster.ca (H.J.); yoellh@mcmaster.ca (H.Y.)

**Keywords:** *Aspergillus*, arctic, microsatellite genotyping, population genetics, triazole resistance, global population structure

## Abstract

*Aspergillus fumigatus* is a saprophytic mold and an opportunistic pathogen with a broad geographic and ecological distribution. *A. fumigatus* is the most common etiological agent of aspergillosis, affecting over 8,000,000 individuals worldwide. Due to the rising number of infections and increasing reports of resistance to antifungal therapy, there is an urgent need to understand *A. fumigatus* populations from local to global levels. However, many geographic locations and ecological niches remain understudied, including soil environments from arctic regions. In this study, we isolated 32 and 52 *A. fumigatus* strains from soils in Iceland and the Northwest Territories of Canada (NWT), respectively. These isolates were genotyped at nine microsatellite loci and the genotypes were compared with each other and with those in other parts of the world. Though significantly differentiated from each other, our analyses revealed that *A. fumigatus* populations from Iceland and NWT contained evidence for both clonal and sexual reproductions, and shared many alleles with each other and with those collected from across Europe, Asia, and the Americas. Interestingly, we found one triazole-resistant strain containing the TR_34_ /L98H mutation in the *cyp51A* gene from NWT. This strain is closely related to a triazole-resistant genotype broadly distributed in India. Together, our results suggest that the northern soil populations of *A. fumigatus* are significantly influenced by those from other geographic regions.

## 1. Introduction

*Aspergillus fumigatus* is a thermotolerant ascomycete mould with a ubiquitous presence around the world. Its primary ecological niche is within decaying plant matter and soil. However, it is also a common opportunistic fungal pathogen capable of infecting both immunocompetent and immunocompromised humans. Over the past three decades, there have been a rising number of reports of antifungal-resistant *A. fumigatus* strains, which have significantly impacted the treatment of patients with *A. fumigatus* infections [1,2,3,4,5]. *A. fumigatus* infections, collectively known as aspergillosis, encompass a range of illnesses from asthma to invasive aspergillosis. Invasive aspergillosis, the most severe form of aspergillosis, is estimated to occur at over 200,000 cases annually worldwide [6]. Depending on the underlying conditions of patients and the effectiveness of antifungal management, the mortality of invasive aspergillosis ranges from 40% to 90%. For both treatment and prophylaxis, triazole antifungals such as itraconazole, voriconazole, posaconazole, and isavuconazole are often used for frontline therapy. Triazole antifungals target the enzyme lanosterol 14α-demethylase encoded by the gene *cyp51A*. This enzyme is required for the biosynthesis of ergosterol, an essential sterol in the cytoplasmic membrane of fungal cells. Resistance to triazoles is commonly conferred by mutations within the *cyp51A* gene, inhibiting triazole binding and/or causing overexpression of the enzyme [7]. However, non-*cyp51A* mediated triazole-resistance mechanisms also exist, such as the overexpression of *abc* and *cdr* efflux pumps [8]. Aside from the triazoles, two other antifungal classes, the polyene amphotericin B and the echinocandins, are used for salvage therapy of aspergillosis, especially in the case of triazole-resistance [9].

Over the past 30 years, there have been rising incidences of triazole resistant *A. fumigatus* infections worldwide, including the identifications of triazole-resistant strains in triazole naïve patients [10,11,12,13]. These results suggest the importance of environmental populations of *A. fumigatus* to patients and to the clinical populations of this species. Consequently, it is extremely important to understand the environmental populations of *A. fumigatus*. Indeed, an increasing number of environmental populations from different geographic regions have been surveyed to aid in monitoring drug resistance rates and identifying/tracking resistant *A. fumigatus* genotypes. The results so far suggest that agricultural use of triazole fungicides can contribute to the development of triazole resistant strains, which subsequently infect patients [14,15,16,17,18]. In these and other studies, a panel of nine highly polymorphic short tandem repeat (STR) loci are often used to analyze the relationships among strains and populations. Based on genotype information at these nine loci on thousands of *A. fumigatus* isolates, divergent lineages as well as high levels of gene flow between geographic populations have been identified [18,19,20]. For example, Ashu et al. [19] revealed eight genetic populations in their global sample with all eight genetic populations being broadly distributed across multiple countries and continents. However, the samples from Cameroon in central western Africa was genetically unique, different from those analyzed so far from Eurasia, Oceania, and the Americas [18].

Among the currently analyzed geographic populations of *A. fumigatus*, almost all have come from tropical, subtropical, and temperate regions. Little is known about geographic populations of *A. fumigatus* from cold climates. In this study, we isolated and analyzed strains of *A. fumigatus* from two high latitude regions: Iceland and the Northwest Territories (NWT) in Canada. Due to their long-distance separation, we hypothesized that populations of *A. fumigatus* from these two geographic regions should be genetically different from each other. In addition, due to their unique climates, we expect that these two *A. fumigatus* geographic populations would be genetically different from those reported from other geographic regions in temperate, subtropical, and tropical climates. Finally, since both geographic regions have very limited agriculture and no history of agricultural fungicide usage, we expected that all isolates from the soil in both regions should be susceptible to the common clinical triazole drugs itraconazole and voriconazole. Our results showed that some of the expectations were met. However, unexpected results were also identified.

## 2. Materials and Methods

### 2.1. Soil Collection, A. fumigatus Isolation, and STR Genotyping

Soil samples were obtained from Iceland and the Northwest Territories in Canada. All soil samples were obtained from at least 1 cm below the soil surface. In Iceland, a total of 314 soil samples were collected from the following six sites: Dimmuborgir (60), Thingvellir (51), Skaftafell (52), Myvatn Lake (42), Landbrotalaug (60), and Reykjavik University (49). In Northwest Territories (NWT) in Canada, 220 soil samples were obtained from the following four sites within and near Yellowknife: Yellowknife Downtown (80), 30 km North of Yellowknife (50), 30 km Northwest of Yellowknife (60), and in Yellowknife Old Town (30). The numbers in parenthesis represent the number of soil samples taken from each site.

To isolate *A. fumigatus*, approximately 0.2 g of soil from each soil sample was put in 1 mL of Sabouraud dextrose (SD) broth containing 50 mg/L chloramphenicol within a 1.5 mL microcentrifuge tube. The soil suspension was mixed through vertexing and incubated at 50 °C for 3 days to select for *A. fumigatus* growth. For the soil samples that failed the initial attempt, two additional attempts were made, each with a different incubation temperature, at 42 °C and 30 °C, respectively. After incubation, mycelia grown on the surface of the broth from each incubation temperature was then transferred to SD agar containing 50 mg/L chloramphenicol and incubated at 37 °C for 3 days. Colonies resembling *A. fumigatus* were sub-cultured to a new agar medium and their conidia were harvested with sterile 30% glycerol and stored at −80 °C. To confirm their identity, these putative *A. fumigatus* isolates were genotyped at the mating type locus using *A. fumigatus* species-specific and mating-type specific primer pairs [21]. Strains confirmed as *A. fumigatus* were then analyzed for their genotypes at nine highly polymorphic short tandem repeat (STR) loci specific for *A. fumigatus*, following protocols previously described by De Valk et al. [22]. The only modification was the fluorescent tag for primers STRAf2C, STRAf 3C, and STRAf 4C that was changed from the TET probe to ATTO550 (IDT) to provide better fragment size detection and scoring. STR fragments at the nine loci were separated using capillary electrophoresis at McMaster University’s MoBix Lab and analyzed using the STR analysis software Osiris [23].

### 2.2. Allelic and Genotypic Diversities

Allelic diversity was calculated for each of the nine STR loci within both the Iceland and NWT populations using the Excel add-in *GenAlEx 6.5* [24,25]. *GenAlEx 6.5* was also used to compute unbiased haploid genetic diversity (uh). The uh diversity measures the probability that two individuals will be different within the population using the sum of allele frequencies within the populations and adjusts for differences in sample size. Additionally, the number of effective alleles (Ne) was calculated. To determine whether the Iceland and NWT populations differ significantly in their mean uh and mean Ne, two-tailed Student’s *T*-tests were conducted.

### 2.3. Clonality and Recombination

To assess the potential evidence for clonality and/or recombination in these two regional populations, we conducted two tests. The first test was the overall linkage disequilibrium within each of the two geographic populations using the standardized index of association ($\bar{r_{d}}$) and the second was the proportion of loci that are phylogenetically compatible. The null hypothesis of the index of association test is random recombination. The function *poppr* from the R package *poppr* was used to calculate $\bar{r_{d}}$ [26]. Statistical significance was determined through 999 permutations. To account for the influence of clonal genotypes on $\bar{r_{d}}$, both populations were clone-corrected using the *poppr* function *clonecorrect* and the $\bar{r_{d}}$ was recalculated. The proportion of phylogenetically compatible pairs of loci was calculated using the software MULTILOCUS v1.3b [27]. Two loci are phylogenetically compatible if all observed genotypes can be accounted for by mutation alone without inferring homoplasy or recombination. This test is also called the four-gamete test. Statistical significance was determined through 1000 randomizations.

### 2.4. Genetic Relationships among Strains from the Two Arctic Populations

To assess the proportion of genetic variation present within and between the two arctic populations, analysis of molecular variance (AMOVA) was conducted using *GenAlEx 6.5* [24,25]. In a panmictic population, most of the variance observed will arise within the samples. In contrast, a high-level among-sample variance would suggest the presence of genetically differentiated populations. The null hypothesis for AMOVA is that there is no genetic difference between the populations tested. For genetic distance calculation among strains, the parameter haploid-SSR was used to incorporate the stepwise mutation model. The smaller the difference between allele repeat numbers at each locus, the smaller the genetic distance between the alleles and consequently, their multilocus genotypes (MLGs) would be more similar to each other. Genetic differentiation between the two arctic populations was calculated using ϕ_pt_, an analogue of F_st_. Significance was determined through 999 permutations. 

To visualize genetic distance between MLGs from Iceland and NWT, a minimum spanning network (MSN) was generated by calculating Bruvo’s genetic distance between strains using the R package *poppr* [26]. Bruvo’s genetic distance is specific for STR genotypes and incorporates the stepwise mutation model. Genetic distance was calculated for each pair of MLGs, and the resulting matrix is visualized in the MSN. Minimum edge genetic distance was set to 0.05. In addition, a multivariate analysis, Discriminant Analysis of Principal Components (DAPC), implemented by *adegenet* package in R, was used to cluster MLGs genotypes in relation to their geographic origins [28]. In this analysis, DAPC first transforms the data using principal component analysis (PCA) to reduce the number of variables and allowing retention of the variables that have the greatest contribution to the variation within the dataset. This is followed by discriminate analysis (DA) to cluster the MLGs by optimizing the between-group variation and reducing within-group variation. 

### 2.5. Genetic Relationships between Arctic Samples and Those from Other Regions

To compare our arctic STR dataset with those from other geographic and climatic regions, STR data from 12 populations within Eurasia, North America, and Oceania, were included, totaling 2339 MLGs [19,29]. Specifically, the number of MLGs within these 12 populations were: Belgium (108), Hamilton, Canada (195), Cameroon (51), France (66), Germany (85), India (94), Netherlands (1082), Norway (203), New Zealand (104), Spain (180), Switzerland (70), and USA (101). 

In our comparisons, the number of private alleles that were present only in Iceland and NWT populations but were absent in these 12 populations was determined. To analyze the level of differentiation between these two arctic populations and those from other geographic regions, we used the same parameters and AMOVA function as described above. In addition to the overall AMOVA, pairwise population ϕ_pt_ was estimated among the 14 geographic populations within this dataset. Statistical significance was determined through 999 permutations. An MSN was generated and DAPC was conducted as described in the above section.

### 2.6. Triazole Susceptibility Testing

Antifungal susceptibility testing was conducted following the reference protocols described in the Clinical and Laboratory Standards Institute (CLSI) document “M38 Reference Method for Broth Dilution Antifungal Susceptibility Testing of Filamentous Fungi” 3rd edition [30]. Here, two triazoles, itraconazole and voriconazole, were used. Two *Candida* strains, *C. parapsilosis* ATCC^®^ 22019 and *C. krusei* ATCC^®^ 6258, were used as references. Briefly, spore suspensions were adjusted to an optical density between 0.09 and 0.13 at 530 nm. The adjusted spore suspensions were then diluted 50× into RPMI1640 at pH 7 ± 0.1. The two triazole antifungals were dissolved to 200 mg/L in DMSO. A two-fold serial dilution series was conducted for each antifungal to create a 3 to 200 mg/L concentration range in addition to a DMSO only control. Each concentration was then diluted 50× in RPMI 1640. Lastly, 100 μL from both the spore suspension and triazole solutions were added to 96-well cell culture plates to test the susceptibility of each isolate from NWT and Iceland at the final drug concentrations 0.03 to 2 mg/L. The 96-well cell culture plates were then incubated at 35 °C without agitation. The minimum inhibitory concentrations (MIC) for both antifungals were visually determined after 48 h. Strains that grew at 2 mg/L were further tested at the antifungal concentrations between 0.25 to 16 mg/L.

### 2.7. Cyp51A Sequencing

The *cyp51A* gene was sequenced for a strain that had an itraconazole and/or voriconazole MIC of 2 or higher to identify putative triazole resistance mutation(s) in this gene. Following the PCR protocol described by Mellado et al. [31], five pairs of primers were used to amplify the *cyp51A* region and its 5′UTR. Amplified PCR products were sent for Sanger sequencing at McMaster University’s MoBix Lab. To identify mutations within the *cyp51A* gene, the obtained sequences were aligned to the *cyp51A* gene from the *A. fumigatus* reference strain af293 using the program MEGA-X [32].

## 3. Results

### 3.1. Isolation Rates of A. fumigatus from Arctic Soils

In total, 52 (23.6%) and 32 (10.2%) *A. fumigatus* isolates were obtained from 220 NWT and 314 Iceland soils samples, respectively. In both regions, there were considerable variations among sites in their isolation rates of *A. fumigatus* (Table 1). Following isolation, all isolates were genotyped at nine microsatellite loci.

### 3.2. Local Genetic Diversity within Iceland and NWT

The STR genotypes were obtained for isolates from both Iceland and NWT *A. fumigatus* populations. Because of the small sample sizes and unequal sample size distributions among local populations from within both Iceland and NWT (Table 1), we elected to not compare the local populations within each of the two regions but instead focused on comparing the two arctic populations. We found that the two regional populations had similar *uh* between Iceland and NWT where no significant difference was observed (Table 2; *p* = 0.36). For the effective number of alleles, the NWT population had a higher value than that from Iceland, but the difference was statistically insignificant (*p* = 0.13). Similarly, the number of observed alleles was higher in the NWT population but after being adjusted for sample size effects, the difference was statistically nonsignificant (*p* = 0.08). Together, the results indicated similar levels of allelic and genotypic diversities between the Iceland and NWT soil populations of *A. fumigatus*. 

### 3.3. Clonality and Recombination within Iceland and Northwest Territories

To investigate evidence for clonality and recombination within the two arctic *A. fumigatus* populations, the $\bar{r_{d}}$ and the proportion of phylogenetically compatible loci were calculated for each population. The $\bar{r_{d}}$ values of Iceland and NWT were 0.36 (*p* = 0.001) and 0.16 (*p* = 0.001), respectively, rejecting the null hypothesis of random mating. The hypothesis of random mating was rejected even after clonal corrections where only 20 and 40 unique MLGs within Iceland and NWT populations, respectively, were retained for analyses. Specifically, after clone-correction, the $\bar{r_{d}}$ values of the Iceland and NWT populations were 0.19 (*p* = 0.001) and 0.13 (*p* = 0.001), respectively. However, phylogenetic compatibility analyses revealed that 55.6% (*p* = 0.001) and 5.6% (*p* = 0.001) pairs of loci within Iceland and NWT were phylogenetically compatible. The high proportions of phylogenetically incompatible pairs of loci, at 44.4% and 94.4%, respectively, for the Iceland and NWT populations, are consistent with recombination. Overall, these results indicate that both the Iceland and NWT soil populations of *A. fumigatus* contain signatures of both clonal and recombining modes of reproduction.

### 3.4. Relationships between Iceland and NWT Samples

**Figure 1:**
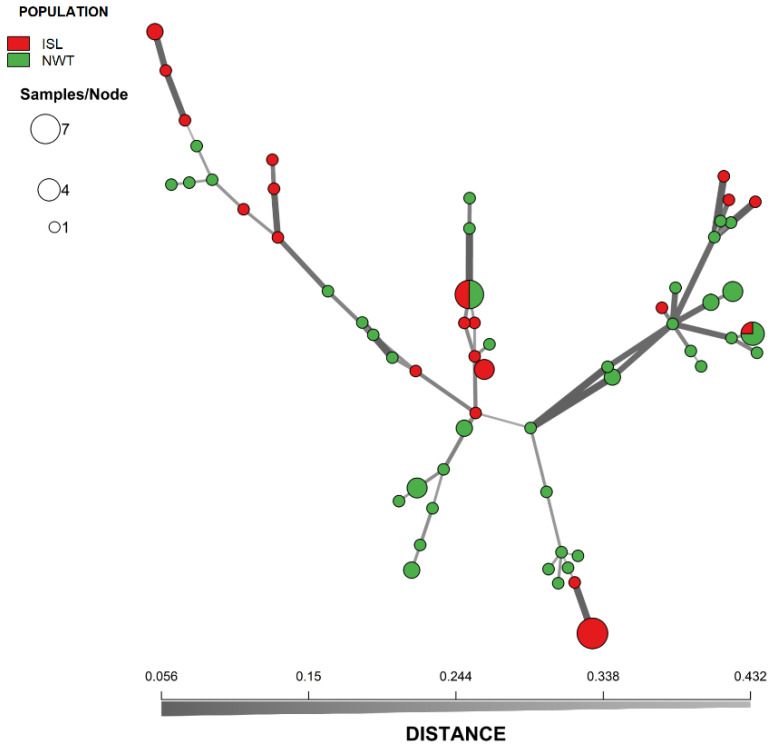
Minimum-spanning network showing the genetic relationship between MLGs of *A. fumigatus* from Iceland and Northwest Territory in Canada. The genetic distance between MLGs was calculated using Bruvo’s genetic distance from the nine microsatellite loci that incorporates the stepwise mutation model. Each node represents one or more identical MLGs, where node size corresponds to the number of strains for each MLG. Nodes that are more genetically similar have darker and thicker edges, whereas nodes genetically distant have lighter and thinner edges.

**Figure 2:**
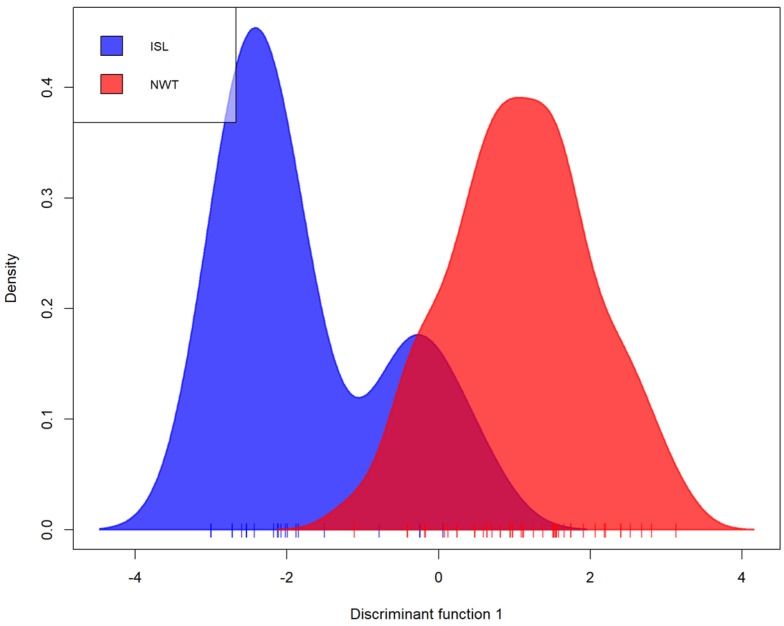
Discriminant analysis of principal components (DAPC) of Iceland and NWT *A. fumigatus* populations representing the first discriminant function in an individual density plot. Isolates were genotyped at nine microsatellite loci and clone corrected, totaling 60 multilocus genotypes. NWT—Northwest Territories, ISL—Iceland.

We analyzed the relationships among *A. fumigatus* isolates from Iceland and NWT. Bruvo’s genetic distance was calculated between isolates of both populations and visualized through an MSN (Figure 1). Overall, while we observed some geographic clustering among MLGs, isolates from the two arctic populations were intermixed. For example, two different MLGs were shared between Iceland and NWT populations. One of these two MLGs was shared among six strains with three isolates from each of the two regional populations. The other was shared among four isolates (one from Iceland and three from NWT). A DAPC of the Iceland and NWT samples yielded comparable results, which both showed some geographic clustering as well as overlaps between these two regions (Figure 2). Results from AMOVA indicated that while the majority (86.8%) of the genetic variations were found within the two regional populations, 13.2% of the total observed genetic variance was found between these two populations (ϕ_pt_ = 0.132, *p* = 0.001). Together, these results are consistent with an overall statistically significant genetic differentiation between these two arctic populations.

### 3.5. Relationship between the Arctic Populations to Additional Global A. fumigatus Populations

To determine how the arctic populations fit in the global context, the allelic and genotypic diversities of 12 other geographic populations totaling 2339 *A. fumigatus* MLGs were compared to the Iceland and NWT *A. fumigatus* populations. Within the 2423 MLGs, the number of private alleles present only within Iceland and NWT was 2 and 1 respectively. In Iceland, these two private alleles were #116 and #117 at the 3A locus. In NWT, the private allele was #57 at the 3B locus. Together, the allelic distribution results suggest limited novel genetic diversity in the arctic soil samples.

To determine the genetic relationships between the 2 arctic populations and the 12 other geographic populations, ϕ_pt_ was calculated between all pairwise population combinations (Table 3). For this analysis, the dataset was also clone-corrected to remove the influence of multiple clonal MLGs in determining genetic differentiation between populations. Overall, our analyses revealed that the two arctic populations of *A. fumigatus* were significantly different from most other geographic populations (Table 3). Specifically, before clonal correction, only the Belgian and French populations showed insignificant difference to the Iceland population. After clonal correction, only the Indian and French populations were not significantly differentiated from the Iceland population while the Norwegian and New Zealand populations were not significantly different from the NWT population.

**Figure 3:**
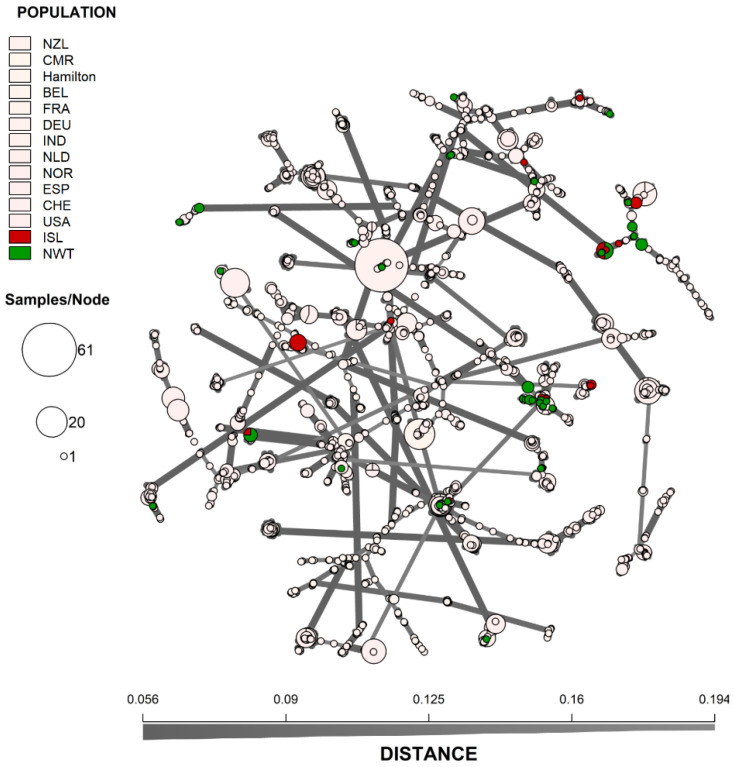
Minimum-spanning network showing the genetic relationship between MLGs from Iceland and Northwest Territory to 12 other *A. fumigatus* geographic populations. The genetic distance between MLGs was calculated using Bruvo’s genetic distance based on the nine microsatellite loci that incorporates the stepwise mutation model. Each node represents one or more identical MLGs, where the node size corresponds to the number of identical MLGs. Nodes that are more genetically similar have darker and thicker edges, whereas nodes genetically distant have lighter and thinner edges. NWT—Northwest Territories, ISL—Iceland, CMR—Cameroon, CAN—Hamilton, Ontario, Canada, BEL—Belgium, FRA—France, DEU—Germany, IND—India, NLD—Netherlands, NOR—Norway, NZL—New Zealand, ESP—Spain, CHE—Switzerland, and USA—United States.

**Figure 4:**
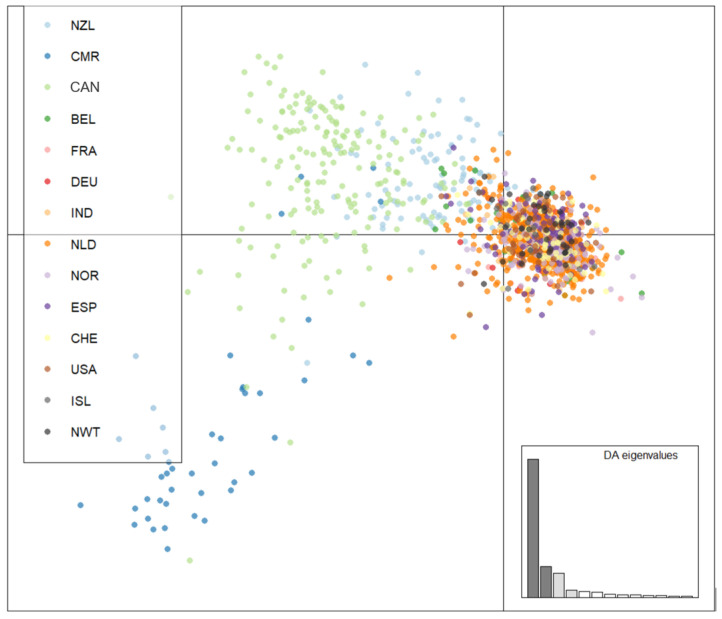
Genetic clustering using discriminant analysis of principal components (DAPC) of Iceland, NWT, Eurasian, North American, and Oceanian *A. fumigatus* populations. Isolates were genotyped at nine microsatellite loci and clone corrected, totaling 1703 unique multilocus genotypes. Populations were clustered according to population of origin. NWT—Northwest Territories, ISL—Iceland, CMR—Cameroon, CAN—Hamilton, Ontario, Canada, BEL—Belgium, FRA—France, DEU—Germany, IND—India, NLD—Netherlands, NOR—Norway, NZL—New Zealand, ESP—Spain, CHE—Switzerland, and USA—United States.

We also analyzed the genotypic similarities among individual MLGs from the 14 geographic populations. An MSN of all 2416 strains was generated to visualize the genetic distance among the MLGs (Figure 3). To highlight the distribution of the arctic isolates, the 12 previously surveyed *A. fumigatus* populations were coloured white. Overall, while the MLGs from the arctic regions showed some geographic clustering, these arctic genotypes were broadly embedded in the global genotype network. This overall pattern was similarly supported by DAPC analyses (Figure 4) where MLGs from Iceland and NWT clustered with those from Eurasia and the United States but were different from the Hamilton, Ontario, Canada population as well as from the Cameroonian population.

### 3.6. Susceptibility Testing

**Figure 5:**
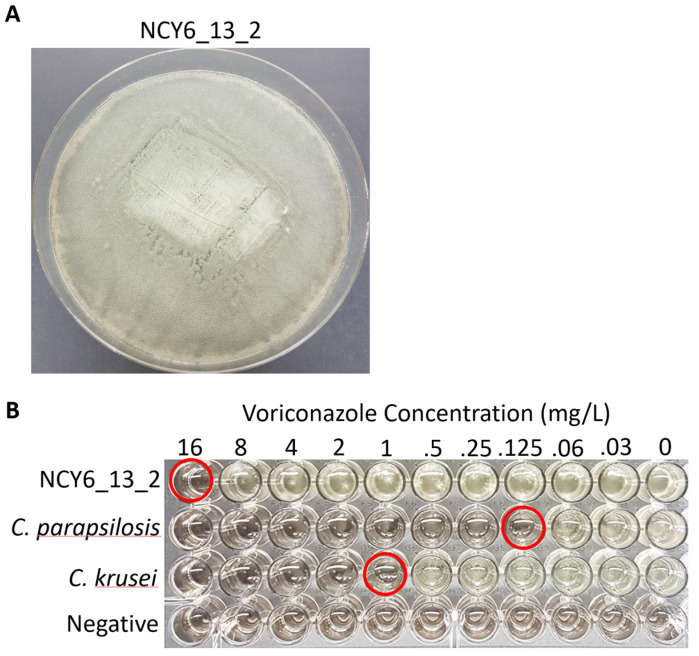
Radial growth and voriconazole susceptibility of triazole resistant *A. fumigatus* strain NCY6_13_2. (**A**) Growth morphology of *A. fumigatus* strain NCY6_13_2 on Malt extract agar after 5-day incubation at 35°C. (**B**) Voriconazole susceptibility of NCY6_13_2. Two control strains, *Candida parasilosis* ATCC^®^ 22019 and *C. krusei* ATCC^®^ 6258, were used to verify the susceptibility results. A negative control that was not inoculated with any microbial strain was included in the bottom row. Susceptibility endpoints are circled in red for each strain.

Antifungal susceptibility testing using voriconazole and itraconazole was conducted for all isolates from the two arctic populations (Table 4). One isolate resistant to both triazoles was identified within NWT but no resistant strain was identified from Iceland (Figure 5). Our sequence analysis revealed that this triazole-resistant strain NCY6_13_2 had the TR_34_/L98H mutation within the *cyp51A* gene. Interestingly, this strain had a near identical genotype at the nine STR loci to a clonal population of triazole-resistant strains discovered from India in 2012, differing by only one repeat unit at the 3A locus and with identical alleles at the other eight STR loci (Table 5) [16]. Furthermore, all the *A. fumigatus* isolates in this Indian clone contained the identical TR_34_/L98H mutation within the *cyp51A* gene as the strain from NWT in our sample.

## 4. Discussion

In this study, we obtained and genotyped *A. fumigatus* isolates from two northern climatic regions, Iceland and Northwest Territories, Canada. This is the first study to investigate *A. fumigatus* population structures at high latitudes and cold climates. A total of 32 (20 unique MLGs) and 52 isolates (40 unique MLGs) were obtained from Iceland and NWT, respectively. Both populations contained abundant allelic and genotypic diversities at the nine microsatellite loci, with evidence of recombination within both populations. However, the observed recombination was not due to random mating. Our analyses demonstrated that while Iceland and NWT shared several identical/similar multilocus genotypes, the two regional populations were significantly differentiated from each other. Similarly, while many alleles were shared, these two arctic soil populations were significantly differentiated from geographic samples from other parts of the world. Interestingly, one triazole resistant strain was identified from NWT and this strain had a near identical MLG as the clonal triazole-resistant population previously identified in India. Below we discuss the implications and relevance of this work to previous studies.

The high level of allelic and genotypic diversity observed within both the Iceland and NWT populations resemble that seen within most regional *A. fumigatus* populations [18,29,33,34,35]. The high allelic and genotypic diversities within both populations (Table 2) suggested that either these two populations were ancient or that there had been multiple migrations from other regions to these two arctic regions. Indeed, the high allelic and genotypic diversities in both regions suggested no evidence of recent selective sweep in either Iceland or NWT. In comparison, a large and broadly distributed MLG within India was found to be caused by a selective sweep of a highly drug-resistant genotype [16]. However, several MLG were over-represented by two or more isolates in both arctic samples, suggesting that local selection could happen in both populations. At present, due to the relatively small and uneven population sizes among local populations within each of these two regions, it is difficult to determine what selective force(s) might be responsible for their over-representation. The over-representation of certain genotypes is consistent with clonal reproduction of *A. fumigatus* in nature. However, evidence for recombination was also found in both regional populations. Our results are similar to those reported previously for other regional populations of *A. fumigatus* [18,29,33,34,35] as well as other human pathogenic and non-pathogenic fungi [36].

Within individual fungal species, both latitudinal differences and geographic distances have been found to correlate with population genetic differences [37,38]. For example, a latitudinal gradient-based differentiation was observed among populations of the chili pepper anthracnose pathogen *Colletotrichum acutatum* [37]. In the rust fungus *Melampsora laricipopulina*, a gradual dispersal of genetic elements across the European populations was observed, which led to higher genetic variations between populations at greater geographic distances [38]. In contrast, the significant genetic differentiation of the Canadian and Icelandic populations of *M. laricipopulina* was likely caused by founder effects [38]. Regarding *A. fumigatus*, geographic *A. fumigatus* populations have been shown to be shaped by both historical differentiation and high contemporary gene flow due to anthropogenic influences [19,29]. Our results from the Icelandic and NWT samples of *A. fumigatus* are consistent with previous findings. Specifically, we observed both allele and genotype-sharing between these two regions but also found an overall significant genetic differentiation both between these two arctic samples as well as between the arctic samples and the geographic populations from other regions of the world. 

At present, while the close relationship between the NWT and Norwegian samples might be due to similar selection pressure, the reasons for the low/insignificant level of differentiation between Icelandic *A. fumigatus* population and those from Belgium, France, and Indian populations as well as between the NWT *A. fumigatus* population and the New Zealand population are unknown. As previously discussed by Korfanty et al. [29], two types of factors may contribute to gene flow between distant countries. The first type is natural factors such as wind. However, given the geographic distance between most of these areas, while possible, direct wind-aided dispersal of *A. fumigatus* conidia is unlikely, especially across hemispheres such as between New Zealand and NWT in Canada (~12,694 km). The second type is anthropogenic factors such as human travel and commercial trade. For both Iceland and NWT, the European Union is a major exporter and importer of goods to and from both regions, contributing to 30% of exports from NWT and 53% of Iceland’s total trade in goods in 2019 [39,40]. Similarly, India was the top importer of NWT goods in 2019, where Asian specific exports from NWT began to increase in 2011. Within Iceland in 2019, 682,700 tourists (34.4%) originated from the European Union [41]. Similarly, Yellowknife in NWT has been a popular destination for observing northern lights, which have attracted tourists from around the world, including from many Asian countries such as China and India. However, the number of tourists from all destinations declined substantially since the start of the SARS-Covid-19 pandemic. Given the resilience of *A. fumigatus* spores to a variety of environmental stressors and their abundance within the atmosphere [42], spores could have followed these anthropogenic routes and migrated to and from Iceland and NWT.

One triazole resistant *A. fumigatus* strain was identified in the NWT population. This triazole-resistant strain contained the TR_34_/L98H mutation in the *cyp51A* gene. This mutation has been reported from environmental and clinical samples from many countries [12,43,44,45], including the selective sweep observed in a widespread Indian genotype of *A. fumigatus* in 2012 [16]. The resistant strain identified in our study had a near identical MLG to the Indian population. However, how the strain migrated into the NWT region is unclear. A recent long distance dispersal event may have occurred, which introduced this strain to NWT from India, potentially by an anthropogenic event. Unfortunately, the lack of clinical research on *A. fumigatus* and aspergillosis within both Iceland and Northern regions in Canada makes it difficult to determine the clinical significance of triazole-resistant aspergillosis in both northern regions and the potential relationship between the triazole-resistant strain isolated here from the soil and the clinical strains of *A. fumigatus* in NWT. Systematic analyses of clinical aspergillosis and susceptibility testing of the strains responsible for such infections in both regions are needed in order to assess the frequency of triazole-resistant aspergillosis and the relationship between environmental and clinical populations of *A. fumigatus* in both Iceland and Northern Canada.

## 5. Conclusions

This study investigated the population structure of the opportunistic fungal pathogen *A. fumigatus* obtained from northern climates above 60° latitude. Prior to this study, limited isolation and genotypic data has been described from soil environments in these northern regions [46,47,48]. Here we characterized *A. fumigatus* diversity present within Iceland and Northwest Territories in Canada. Our study revealed significant genetic diversity with evidence of both clonal and sexual reproduction within both regional populations. While evidence for gene flow was found between these two regional arctic populations as well as between them and other geographic populations, unique alleles and genotypes were also found in both arctic populations. The finding of a triazole-resistant strain from the arctic with a nearly identical genotype to strains in India demonstrates the complexity of managing drug resistance in *A. fumigatus*. Our study highlights the importance of both genetically characterizing *A. fumigatus* populations from a diversity of geographic and ecological niches and identifying the level of triazole susceptibility within these populations to help track the spread of drug-resistant strains. Indeed, the discovery of high genetic diversity in cold environments coupled with the known high-temperature tolerance of *A. fumigatus* calls for greater effort for understanding the thermo-adaptability of this organism, with implications even for space travel and contamination of Mars from the Mars missions [46,49].

## Figures and Tables

**Table 1:** Soil samples and *A. fumigatus* isolates obtained from Iceland and Northwest Territories (NWT).

Country/Territory	Local Site	Number of Soil Samples	Number of Isolates (MAT1-1:MAT1-2) *
**Iceland**	Dimmuborgir	60	1 (0:1)
	Thingvellir	51	8 (3:5)
	Skaftafell	52	6 (4:2)
	Myvatn Lake	42	10 (7:3)
	Landbrotalaug	60	1 *
	Reykjavik University	49	6 (4:2)
	Total	312	32 (18:13) *
**Canada/NWT**	Yellowknife Downtown	80	43 (24:17) *
	30 km North of Yellowknife	50	0
	30 km Northwest of Yellowknife	60	3 (0:3)
	Yellowknife Old Town	30	6 (1:5)
	Total	220	52 (25:25) *

* 1 isolate from Iceland and 2 from NWT were unable to be genotyped at the MAT locus.

**Table 2:** Number of alleles and allelic diversity for the nine microsatellite loci of 32 and 52 Aspergillus fumigatus isolates from Iceland and Northwest Territories, respectively.

Population	Locus	N ^1^	Na ^2^	Ne ^3^	Uh ^4^
Iceland	2A	32	7	4.92	0.82
	2B	32	5	4.70	0.81
	2C	32	8	6.10	0.86
	3A	32	13	6.02	0.86
	3B	32	6	4.66	0.81
	3C	32	10	6.24	0.87
	4A	29	5	2.87	0.68
	4B	32	6	2.94	0.68
	4C	30	10	5.63	0.85
	Mean	31.44	7.78	4.90	0.81
	Standard Error	0.38	0.91	0.43	0.03
Northwest Territories	2A	51	14	4.43	0.79
	2B	52	10	3.99	0.76
	2C	52	11	6.93	0.87
	3A	50	19	13.74	0.95
	3B	50	11	4.63	0.80
	3C	50	19	12.89	0.94
	4A	52	9	3.25	0.71
	4B	52	8	3.22	0.70
	4C	52	14	5.88	0.85
	Mean	51.22	12.78	6.55	0.82
	Standard Error	0.32	1.35	1.34	0.03

^1^ N = sample size; ^2^ Na = number of unique alleles; ^3^ Ne = number of effective alleles = 1/(Σp_i_^2^) where p_i_ is the frequency of the ith allele for the population; ^4^ uh = unbiased diversity = (N/(N−1)) × (1−Σp_i_^2^).

**Table 3:** Pairwise differentiations between Iceland and Northwest Territories *A. fumigatus* populations to each other and 12 *A. fumigatus* populations from Eurasia, Oceania, and North America. NWT/ISL row represents the Iceland or Northwest Territories population being compared to the other. NWT—Northwest Territories, ISL—Iceland, CMR—Cameroon, CAN—Hamilton, Ontario, Canada, BEL—Belgium, FRA—France, DEU—Germany, IND—India, NLD—Netherlands, NOR—Norway, NZL—New Zealand, ESP—Spain, CHE—Switzerland, and USA—United States.

Pairwise ϕ_pt_				
	Iceland		Northwest Territories	
Country	Original Dataset	Clone Corrected	Original Dataset	Clone Corrected
NWT/ISL	0.132 **	0.083 *	0.132 **	0.083 *
CMR	0.580 ***	0.629 ***	0.598 ***	0.588 ***
CAN	0.412 ***	0.439 ***	0.337 ***	0.327 ***
BEL	0.020	0.048 *	0.178 ***	0.041 **
FRA	0.028	0.012	0.070 **	0.050 *
DEU	0.170 ***	0.192 ***	0.285 ***	0.206 ***
IND	0.130 ***	0.052	0.213 ***	0.056 *
NLD	0.054 **	0.069 *	0.088 ***	0.062 **
NOR	0.152 ***	0.050 *	0.037 **	0.014
NZL	0.067 **	0.123 **	0.051 **	0.024
ESP	0.082 **	0.074 *	0.122 ***	0.069 ***
CHE	0.089 **	0.100 **	0.063 ***	0.036 *
USA	0.162 ***	0.146 **	0.129 ***	0.051 **

* *p*-value = 0.05, ** *p*-value = 0.01, *** *p*-value = 0.001.

**Table 4:** Triazole antifungal susceptibility of the 32 and 52 *A. fumigatus* isolates from Iceland and NWT, respectively, to itraconazole and voriconazole. Numbers in the table refer to the number of strains with the respective minimum inhibitory concentrations for each of the two drugs in each geographic population.

Country/Territory	Triazole	Minimum Inhibitory Concentration (mg/L)					
		0.125	0.25	0.5	1	2	16
Iceland	Itraconazole	0	0	24	8	0	0
	Voriconazole	2	29	1	0	0	0
Canada/NWT	Itraconazole	0	0	35	16	0	1
	Voriconazole	0	27	24	0	0	1

**Table 5:** Short tandem repeat genotype of the *A. fumigatus* strain with the TR_34_/L98H mutation in the triazole-target gene *cyp51A* obtained from Northwest Territories in Canada. Alleles at eight of the nine STR loci in this strain were identical to those of the large Indian triazole-resistant clonal population as reported by Chowdhary et al. [16].

Strain	Region	2A	2B	2C	3A	3B	3C	4A	4B	4C
NCY6-13_2	NWT	14	20	9	31	8	10	8	10	28
1042/09	India	14	20	9	31	9	10	8	10	28

## Data Availability

Not applicable.
